# Advanced detection of cervical cancer biomarkers using engineered filamentous phage nanofibers

**DOI:** 10.1007/s00253-024-13058-w

**Published:** 2024-02-19

**Authors:** Xu Zhou, Yicun Wang, Meijing Bao, Yuqing Chu, Ruixue Liu, Qi Chen, Yang Lin

**Affiliations:** 1 Department of Obstetrics and Gynecology, The Second Hospital of Jilin University, 218 Ziqiang St, Changchun, 130041 Jilin China; 2 Jilin Provincial Key Laboratory On Molecular and Chemical Genetic, The Second Hospital of Jilin University, 218 Ziqiang St, Changchun, 130041 Jilin China

**Keywords:** Phage, Nanofiber, Cervical cancer, Diagnostic vector, Cancer diagnosis

## Abstract

**Abstract:**

Cervical cancer is a major global health concern, characterized by its high incidence and mortality rates. The detection of tumor markers is crucial for managing cancer, making treatment decisions, and monitoring disease progression. Vascular endothelial growth factor (VEGF) and programmed death-ligand 1 (PDL-1) are key targets in cervical cancer therapy and valuable biomarkers in predicting treatment response and prognosis. In this study, we found that combining the measurement of VEGF and soluble PDL-1 can be used for diagnosing and evaluating the progression of cervical cancer. To explore a more convenient approach for detecting and assessing cervical cancer, we designed and prepared an engineered fd bacteriophage, a human-safe viral nanofiber, equipped with two peptides targeting VEGF and PD-L1. The dual-display *phage* nanofiber specifically recognizes and binds to both proteins. Utilizing this nanofiber as a novel capture agent, we developed a new enzyme-linked immunosorbent assay (ELISA) method. This method shows significantly enhanced detection sensitivity compared to conventional ELISA methods, which use either anti-VEGF or anti-PD-L1 antibodies as capture agents. Therefore, the phage dual-display nanofiber presents significant potential in detecting cancer markers, evaluating medication efficacy, and advancing immunotherapy drug development.

**Key points:**

*• The combined measurement of VEGF and soluble Programmed Death-Ligand 1(sPD-L1) demonstrates an additive effect in the diagnosis of cervical cancer. Fd phage nanofibers have been ingeniously engineered to display peptides that bind to VEGF and PD-L1, enabling the simultaneous detection of both proteins within a single assay*

*• Genetically engineered phage nanofibers, adorned with two distinct peptides, can be utilized for the diagnosis and prognosis of cancer and can be mass-produced cost-effectively through bacterial infections*

*• Employing dual-display fd phage nanofibers as capture probes, the phage ELISA method exhibited significantly enhanced detection sensitivity compared to traditional sandwich ELISA. Furthermore, phage ELISA facilitates the detection of a single protein or the simultaneous detection of multiple proteins, rendering them powerful tools for protein analysis and diagnosis across various fields, including cancer research*

**Graphical Abstract:**

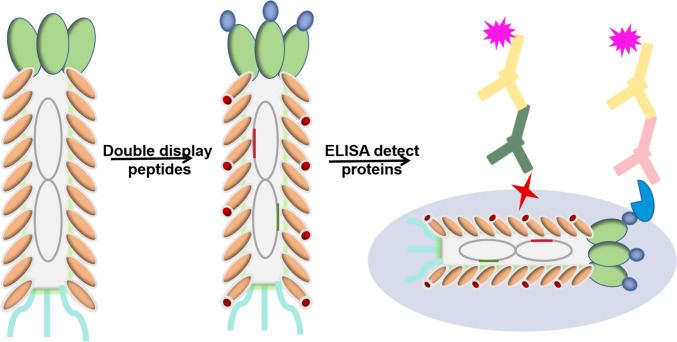

**Supplementary Information:**

The online version contains supplementary material available at 10.1007/s00253-024-13058-w.

## Introduction

Cervical cancer is ranked as the fourth most prevalent cancer among women globally, with an estimated 604,000 new cases and 342,000 deaths reported in 2020 (Arbyn et al. 2020). The primary etiological factor for cervical cancer is infection with *human papillomavirus* (HPV) (Zhen et al. 2023), although its exact pathogenesis remains elusive.

Vascular endothelial growth factor (VEGF) plays a pivotal role in the initiation and progression of cervical cancer (Piškur et al. 2023). Elevated VEGF levels are associated with a poorer prognosis, an increased risk of tumor recurrence, and metastasis in cervical cancer patients (Goncharuk et al. 2009). Consequently, VEGF has been identified as a potential therapeutic target in cervical cancer. Furthermore, VEGF can serve as a biomarker for diagnosing cervical cancer and predicting treatment response and prognosis (Du et al. 2014; Mathur et al. 2005). Compared to healthy individuals, cancer patients often exhibit elevated serum VEGF levels. Elevated serum VEGF levels have been associated with poorer responses to chemotherapy and radiation therapy, as well as an increased risk of disease progression and metastasis (Zhu et al. 2016). As a result, VEGF is of considerable importance both as a detection target and a prognostic indicator in cervical cancer. Programmed death ligand-1 (PD-L1) plays a key role in negatively regulating tumor immune responses by suppressing T lymphocyte activity and is significantly correlated with tumor cell metastasis (Yao et al. 2019).sPD-L1 has been recognized as a diagnostic and prognostic biomarker in various cancer types. In cervical cancer, PD-L1 expression is found to be associated with a poorer prognosis and a higher risk of cancer recurrence and metastasis(Chivu-Economescu et al. 2023; Jovanović et al. 2019; Liu et al. 2022). Solorzano-Ibarra et al. have demonstrated that identifying patients with high levels of sPD-L1 may aid in evaluating antibody therapy (Solorzano-Ibarra et al. 2021).PD-1/PD-L1 immune checkpoint blockade enhances the cellular immune response against cancer. By blocking the interaction between PD-1 on T cells and PD-L1 on cancer cells, immune checkpoint inhibitors restore T cell function and promote tumor cell killing. This leads to an improved anti-tumor immune response, resulting in enhanced tumor control and improved clinical outcomes in cancer patients. PD-L1 serves as an important biomarker and therapeutic target in cervical cancer. Pembrolizumab and nivolumab, drugs targeting the PD-1/PD-L1 pathway (Gaikwad et al. 2022), have been developed and are currently undergoing clinical trials for cervical cancer treatment.

The *fd phage*, a nanowire-like *filamentous phage*, infects bacteria specifically, posing no harm to humans (Pellegri et al. 2023), featuring a diameter of approximately 6.5 nm and a length extending to several micrometers. The virus consists of approximately 2700 copies of a major coating protein, pVIII, distributed along the virus’s length, and five copies each of four minor coating proteins (pIII, pVI, pVII, and pIX) that cap the ends of the virus (Rakonjac et al. 2023, 2017). Phage display, a technique based on the genetic modification of phage DNA, enables the expression of peptides on the phage surface (Huang et al. 2022). Fd phages are widely utilized as vectors for displaying peptides (Cao et al. 2012; Enshell-Seijffers et al. 2001; Hajitou et al. 2007), which can be engineered to specifically bind to targets for applications such as drug discovery, targeted therapy, and detection agents. Combining tumor markers can enhance the effectiveness of clinical diagnosis in cervical cancer (Laengsri et al. 2018; Li et al. 2013; Ran et al. 2021). This study aims to develop a research platform for combined detection reagents. In this study, we modified the fd phage vector to display a combination of two peptides on its surface (Wang et al. 2015). A novel fd phage nanofiber, named fd-LC-WT, has been designed and developed for the simultaneous capture of vascular endothelial growth factor (VEGF) and soluble programmed death-ligand 1 (sPD-L1) in the bloodstream. These nanofibers can be employed for the detection of VEGF, PD-L1, or both, depending on the selected detector antibodies. The engineered phage displaying anti-VEGF and anti-PD-L1 peptides has the potential for application in tumor immunotherapy. Dual blockade of anti-VEGF and anti-PD-L1 enhances tumor immune therapy by inhibiting tumor angiogenesis and promoting T cell activation, resulting in improved anti-tumor immune responses and enhanced tumor control.

We established a phage ELISA detection system based on these nanofibers, which demonstrated higher positivity and specificity compared to conventional sandwich ELISA. No significant difference was observed between the combined protein ELISA measurements and the phage ELISA in capturing the targeted proteins, VEGF and PD-L1. Subsequently, the phage nanofibers were employed to detect VEGF and sPD-L1 in the serum of cervical cancer patients. The findings indicate that *fd phage* nanofibers can be effectively utilized as detection agents to enhance the efficacy of detection and diagnosis, offering a cost-effective approach.

## Materials and methods

### Strains, phage, and ELISA kits

The wild type *phage* (WT phage), dual display phage vector, and *Escherichia coli ER2738* cells were maintained in our laboratory(Wang et al. 2018). Sandwich ELISA kits for VEGF and PD-L1 were obtained from Shanghai Enzyme-linked Biotechnology Company.

## Serum samples

Human serum samples were obtained from the Second Hospital of Jilin University, with approval from the Committee on Medical Ethics of the Second Hospital of Jilin University (Changchun, China). This study enrolled a cohort comprising 71 cervical cancer patients, 25 patients with precancerous cervical lesions (PCL), and 16 healthy individuals. Diagnosis and treatment of these patients occurred in Changchun, Jilin, from September 2020 to March 2023. All participants provided informed consent prior to the collection of their serum samples, which were then stored at − 80 °C until analysis.

## Transfection and production of dual display phage nanofiber

The anti-VEGF peptide (LSTSPHTGGGSC) (Wang et al. 2021; Rezaei et al. 2020) and anti-PD-L1 peptide (WHRSYYTWNLNT) (Liu et al. 2019) were displayed on the major coat protein of fd phages utilizing a previously reported protocol (Wang et al. 2018). Briefly, DNA fragments encoding these peptides were synthesized and cloned into gene VIII and gene III of the phage vector, respectively. Subsequently, the recombinant phages were transformed into *E. coli ER2738 cells.* The expression of the inserted peptide was confirmed by SDS-PAGE using 20% acrylamide gels, visualized with silver staining. The fd-LC-WT dual-display phage nanofibers were analyzed using western blotting. TEM images of fd-LC-WT nanofibers were captured and characterized by using a previously established protocol. To assess the selectivity of fd-LC-WT, western blot analysis for three common cervical cancer biomarkers (CEA, CA125, and PSA) was conducted.

## Optimization of phage ELISA

A phage ELISA for antigen detection was developed utilizing a previously established protocol (Wang et al. 2018). Checkerboard titration alongside sandwich ELISA was used to determine the optimal concentrations of capture and detection antibodies. Briefly, a series of double-display phage as capture antibodies (0, 25, 50, 75, and 100 µg/mL in PBS, 50ul per well) were coated onto a 96-well microplate at 4 ℃ overnight. The microplate was blocked with 2.5% (m/v) skim milk powder in 10 mM PBS at 37 ℃ for 1 h. After washing three times with PBS containing 0.05% Tween 20 (PBST), the VEGF and PD-L1 positive serum was added to the microplate, followed by the addition of an equal volume of mixed antibodies (anti-VEGF and anti-PDL-1 polyclonal antibodies) and incubated at 37 ℃ for 1 h, with three subsequent washes with PBST. Then, 100 µL per well of HRP-conjugated anti-human antibody was added and incubated at 37 ℃ for 1 h, followed by the addition of 100 µL per well of TMB (3,3′,5,5′-tetramethylbenzidine) substrate solution and incubation at 37 ℃ for 10 min. Finally, the reaction was stopped by adding 2 M sulfuric acid, and the absorbance was measured at 450 nm using a microplate reader.

In this experiment, nine serum samples, each tested in triplicate, were used to assess the reproducibility of the phage sandwich ELISA. The coefficients of variation (CVs) for intra-batch and inter-batch assays were calculated to evaluate the consistency of the phage sandwich ELISA.

## Comparisons of the phage ELISA with commercial ELISA kits

**Scheme 1:**
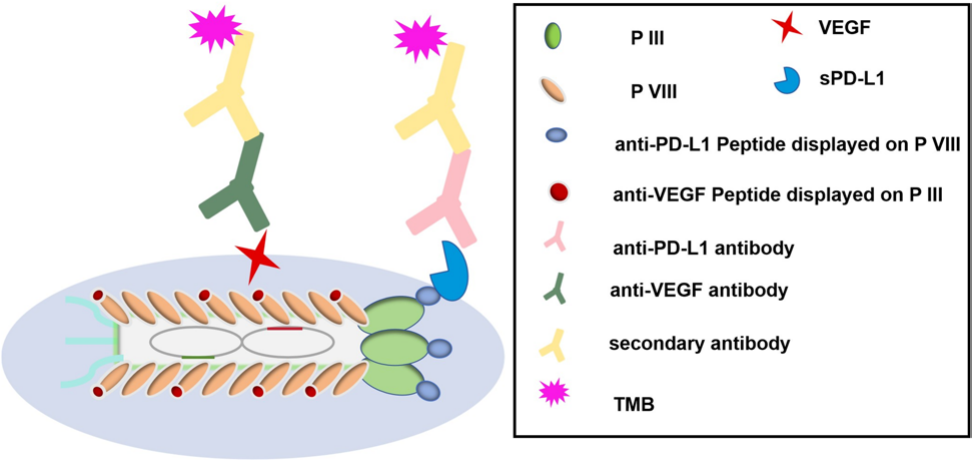
Scheme of phage sandwich ELISA. The general principle of using fd-LC-WT for the detection of VEGF and sPD-L1 in serum is that using fd-LC-WT as capturing antibodies could bind to anti-PD-L1 antibodies and anti-VEGF antibodies

Western blot analysis confirmed the ability of fd-LC-WT to target VEGF and PD-L1. Both VEGF and PD-L1 were denatured in SDS solution and electrically separated on an SDS gel. Subsequently, fd-LC-WT and wild-type phage were utilized as primary antibodies to interact with coating proteins on the blotted membrane. HRP-conjugated anti-phage secondary antibody was allowed to interact with fd-LC-WT. As previously reported (Wang et al. 2015, 2018), healthy human serum samples were used to establish control values, and serum from 71 cervical cancer patients was studied. For detecting serum from 71 cancer patients, commercial ELISA kits were used as controls, while fd-LC-WT was employed as a coating antigen to capture specific proteins. In the phage ELISA method, (as illustrated in Scheme 1) a solution of 50 μg/mL in 0.05 M sodium carbonate buffer (pH 9.6) was used. To detect VEGF, the anti-VEGF antibody was used as a detector antibody to interact with the VEGF in serum. To detect sPD-L1, anti-PD-L1 antibody was used to interact with PD-L1 in serum. To detect both VEGF and PDL1, the mix of anti-PDL1 and anti-VEGF antibody was used to interact with the VEGF and PD-L1 in serum. The mean ± two standard deviations (SDs) from healthy human sample were used as the cutoff value; serum samples were classified as positive when exceeding the cutoff value.

## Statistical analysis

Statistical analysis was conducted using GraphPad Prism software (GraphPad Software, San Diego, CA, USA). Kappa index values were calculated to evaluate the agreement between the phage ELISA and the commercial ELISA kit. These calculations were executed using SPSS software (IBM Corporation).

## Results

### Cervical cancer patients exhibited elevated levels of serum VEGF and sPD-L1

**Fig. 1:**
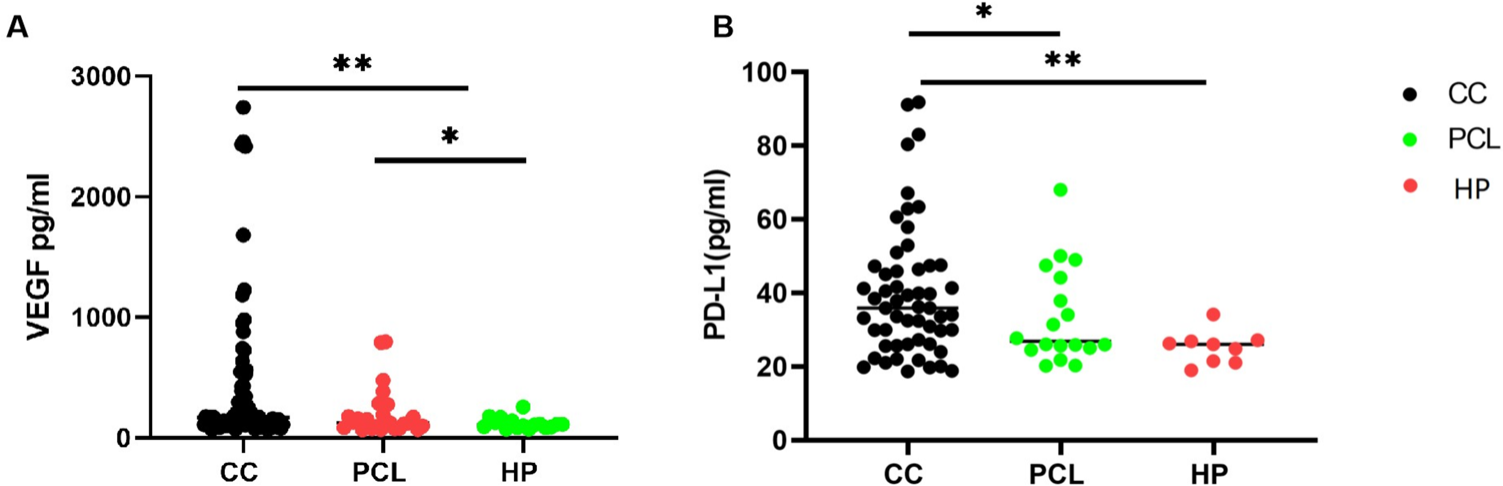
The concentration of VEGF and PD-L1 in the 16 healthy human sera samples (HP), 25 precancerous cervical lesion samples (PCL), and 71 cervical cancer samples (CC) were studied in this article. (**A**) The concentration of VEGF in CC is higher than group PCL and HP; (**B**) the concentration of PD-L1 in CC is higher than in group PCL and HP. (* P < 0.05, ** P < *0.01*)

To evaluate the potential of serum VEGF and sPD-L1 as biomarkers for the diagnosis of cervical cancer, serum levels of these markers were measured using the sandwich ELISA technique. Of the 112 subjects, 71 (63.4%) were cervical cancer patients, 25 (22.3%) had precancerous cervical lesions, and 16 (14.3%) were healthy controls. The study revealed that cervical cancer patients had significantly higher levels of serum VEGF and sPD-L1 compared to those with precancerous cervical lesion and healthy controls (Fig. 1). The mean serum levels of VEGF were 424.3 pg/mL, 206.5 pg/mL, and 118.8 pg/mL in cervical cancer patients, those with precancerous cervical lesions, and healthy controls, respectively. Similarly, the mean serum levels of sPD-L1 were 42.3 pg/mL, 34.2 pg/mL, and 25.4 pg/mL in these groups, respectively.

Data analysis performed with GraphPad Prism revealed that significant differences were observed in the concentrations of VEGF and sPD-L1 between the cervical cancer group and both the precancerous lesion and healthy control groups. Furthermore, a steady increase was noted in the mean serum levels of VEGF and sPD-L1 correlating with the progression of the disease.

### The combined measurement of VEGF and sPD-L1 for diagnosis of cervical cancer

**Fig. 2:**
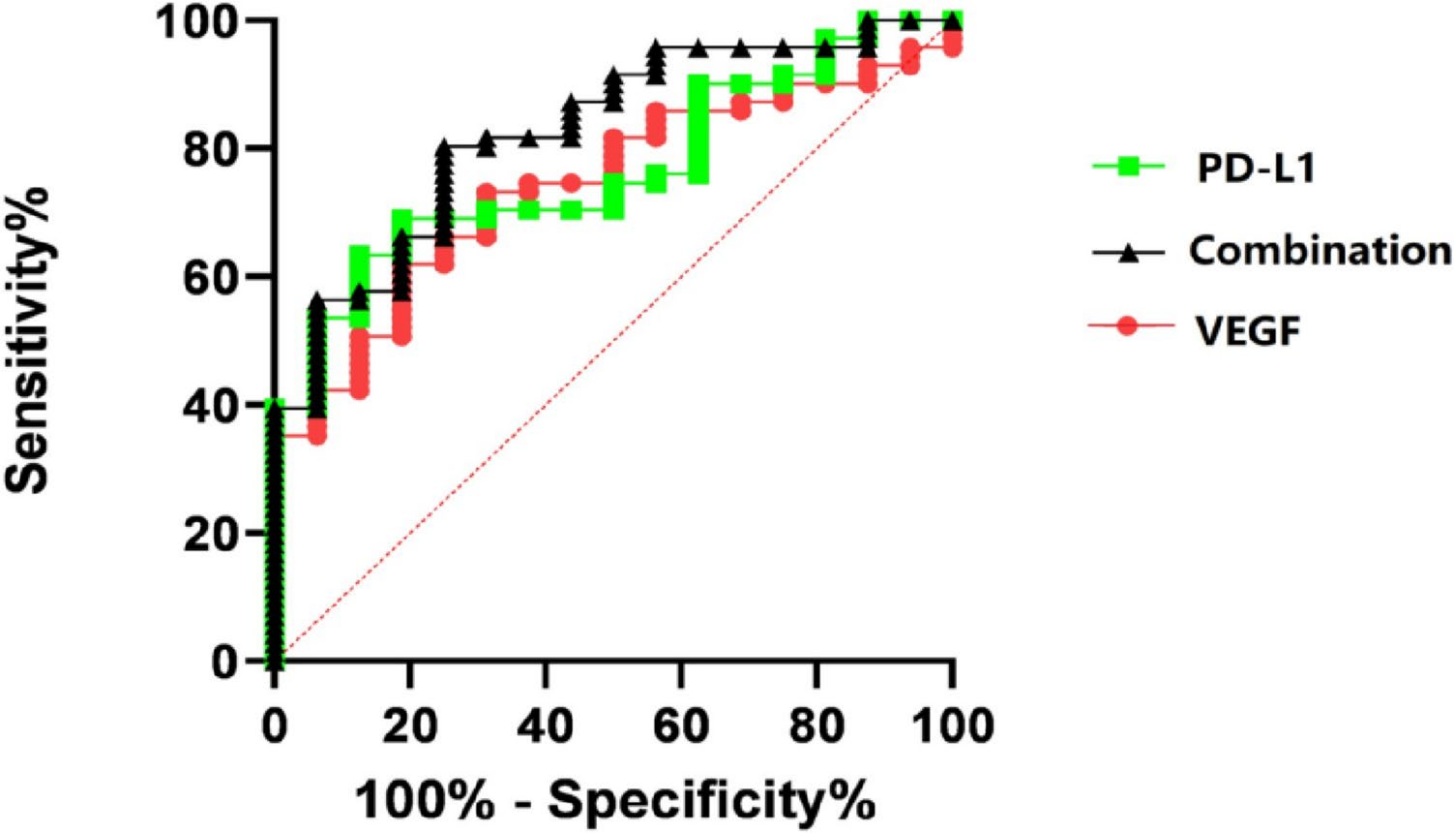
Comparison of receiver-operating characteristic (ROC) curves of the VEGF, PD-L1, and combination measurement of VEGF and PD-L1. The ROC curve of ELISA for identifying cervical cancer in the cohort of 71 patients with cervical cancer and 16 healthy controls. The area under the ROC curve for the combined measurement was 0.826, which was significantly higher than the area under the curve for VEGF alone (0.743) or sPD-L1 alone (0.765). ( ** P < *0.01*)

Further research and clinical trials are necessary to validate the diagnostic efficacy and potential clinical utility of combining sPD-L1 and VEGF measurements for the diagnosis and management of cervical cancer. In this study, the individual and combined diagnostic values of sPD-L1 and VEGF were evaluated. Results indicated that the serum levels of VEGF and sPD-L1 had area under the Receiver Operating Characteristic (ROC) curve (AUC) values of 0.743 for VEGF and 0.765 for sPD-L1, respectively (*P* < *0.01*). When combined, the AUC for serum VEGF and sPD-L1 increased to 0.826 (*P* < *0.01*) (Fig. 2). The sensitivity and specificity of this combined approach were higher compared to using either biomarker alone, suggesting an additive effect in their diagnostic utility.

### Fd nanofibers specifically recognize and bind to VEGF and sPD-L1

**Fig. 3:**
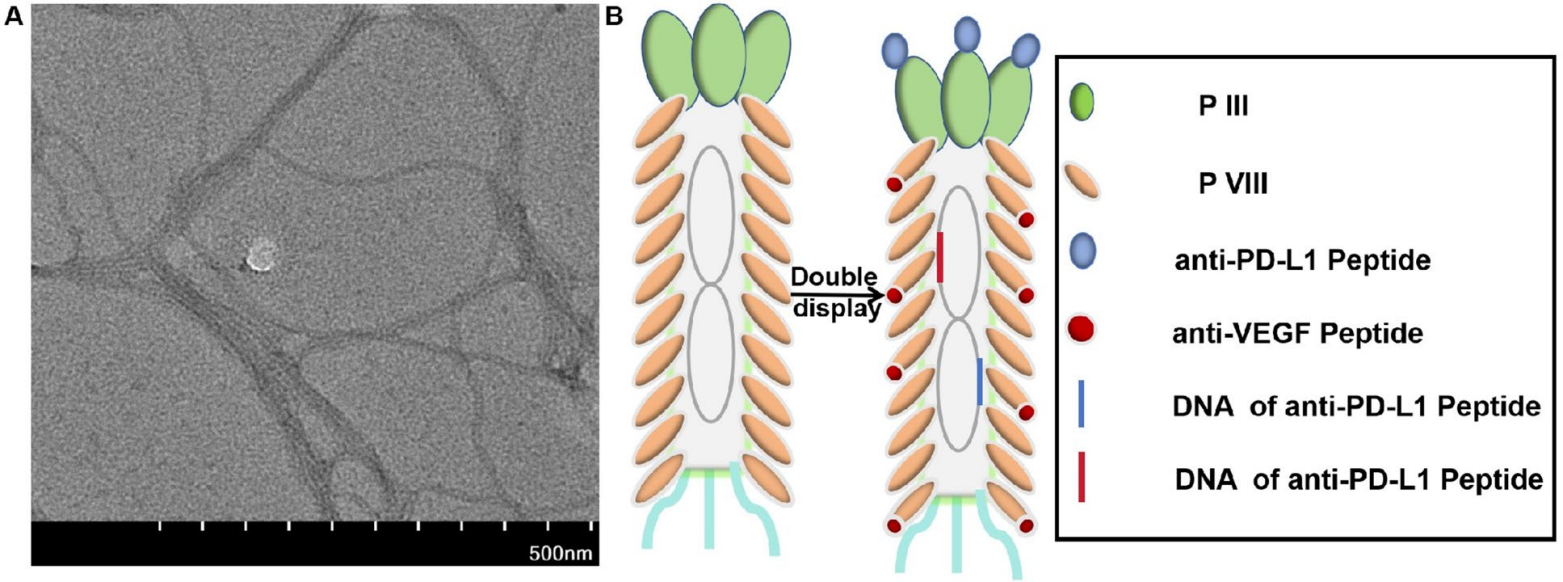
TEM image and scheme of fd-LC-WT nanofibers. **A** This image shows that the fd-LC-WT nanofibers were approximately 900 nm long. **B** pIII and pVIII phage-genome double display system and general principle of establishment of fd-LC-WT binding to VEGF and PD-L1

**Fig. 4:**
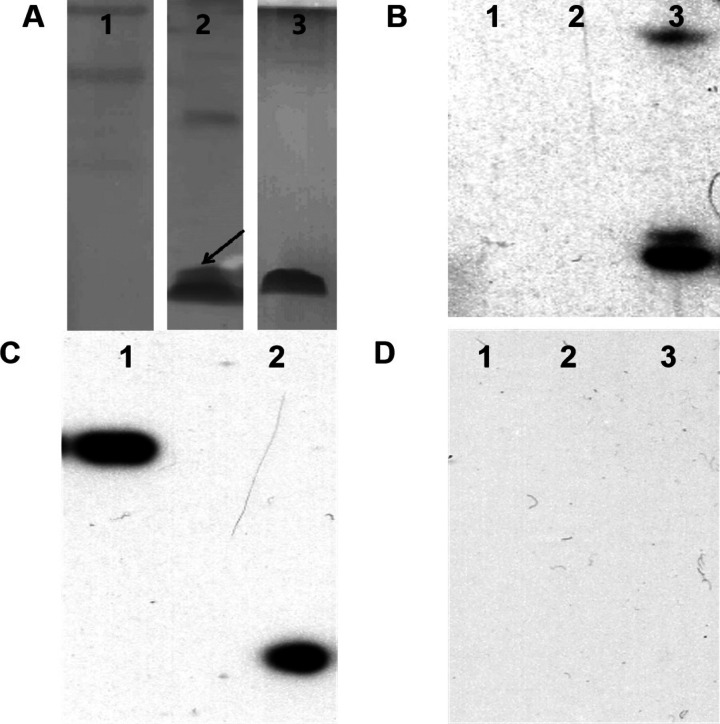
SDS-PAGE analysis of fd-LC-WT. **A** Lane 1 marker, lane 2, fd-LC-WT; lane 3, wild type (WT) phage. **B** Western blotting for fd-LC-WT and WT phage. Lane 1, WT phage with cervical cancer serum; lane 2, fd-LC-WT with PBS; lane 3, fd-LC-WT with VEGF and PD-L1 positive cervical cancer serum. **C** Western blotting for VEGF and PD-L1. Lane 1, PD-L1 with fd-LC-WT; lane 2, VEGF with fd-LC-WT. **D** Western blotting for other cervical cancer biomarkers with fd-LC-WT. Lane 1 CEA; lane 2 CA125; lane 3, PSA

In this study, transmission electron microscopy (TEM) analysis revealed that the dual-displayed phage formed rod-like virus nanofibers (Fig. 3), indicating the successful construction of the modified *fd phage*. Prior to the preparation of the phage proteins, sequencing analysis confirmed the insertion of the DNA fragment encoding the respective peptides. SDS-PAGE analysis additionally confirmed the successful display of the LC and MT peptides on the surface of the fd phage (Fig. 4A). The binding capability of the fd-LC-WT phage to VEGF and PD-L1 is demonstrated in Fig. 4B, C, respectively. Notably, the fd-LC-WT phage exhibited high specificity as it did not recognize other cervical cancer biomarkers, such as CEA, CA125, and PSA (Fig. 4D). This result indicates that Fd-LC-WT phage nanofibers, displaying the anti-VEGF and anti-PD-L1 peptides, specifically recognized VEGF and PD-L1, respectively.

### Establishment of a sandwich ELISA based on phage nanoparticles

The anti-VEGF polyclonal antibody was optimally diluted at 1:5000, the anti-PD-L1 polyclonal antibody at 1:2500, and the optimal concentration of fd-LC-WT phage was determined to be 50 μg/mL (refer to Table 1, Table S1, Table S2). Using these optimized conditions, a sandwich ELISA was successfully established. The coefficient of variation (CV%) for the analysis of nine serum samples ranged from 2.15 to 8.36, while inter-assay CV% for these samples ranged from 6.15 to 11.58 (Table S3, Table S4).

**Table 1:** OD 450 of phage sandwich ELISA checkerboard

Antigen to be detected	Capture antibody: fd-LC-WT	Detection antibody mix1	Mix 2	Mix 3	Mix 4	Mix 5	Mix 6	Mix 7	Mix 8	Mix 9	PBS
Positive: VEGF and PD-L1	25 µg/mL	0.72	0.67	0.69	0.69	0.69	0.70	0.73	0.58	0.49	0.09
	50 µg/mL	0.98	0.99	0.79	0.68	0.7	0.77	0.68	0.55	0.49	0.09
	75 µg/mL	0.93	0.93	0.96	0.89	0.89	0.83	0.79	0.69	0.58	0.09
	100 µg/mL	0.94	0.98	0.92	0.92	0.97	0.99	0.79	0.79	0.57	0.1
Negative:	25 µg/mL	0.25	0.25	0.24	0.24	0.25	0.24	0.27	0.23	0.22	0.10
	50 µg/mL	0.27	0.25	0.24	0.25	0.24	0.23	0.28	0.23	0.21	0.1
	75 µg/mL	0.31	0.28	0.26	0.29	0.27	0.25	0.28	0.25	0.29	0.09
	100 µg/mL	0.27	0.29	0.28	0.25	0.29	0.27	0.32	0.28	0.29	0.11

^*^The VEGF and PD-L1 positive serum and negative serum were used as detected positive and negative antigens in checkerboard, different concentrations of fd-LC-WT were used as capture antibody in checker board, and the mixture of different dilutions of anti- VEGF and anti-PD-L1 antibodies were used as detection antibody

The ELISA checkerboard is for enhancing sensitivity and specificity and ensuring optimal performance of the phage-ELISA in detecting

### Detection of VEGF and sPD-L1 by phage ELISA method

**Fig. 5:**
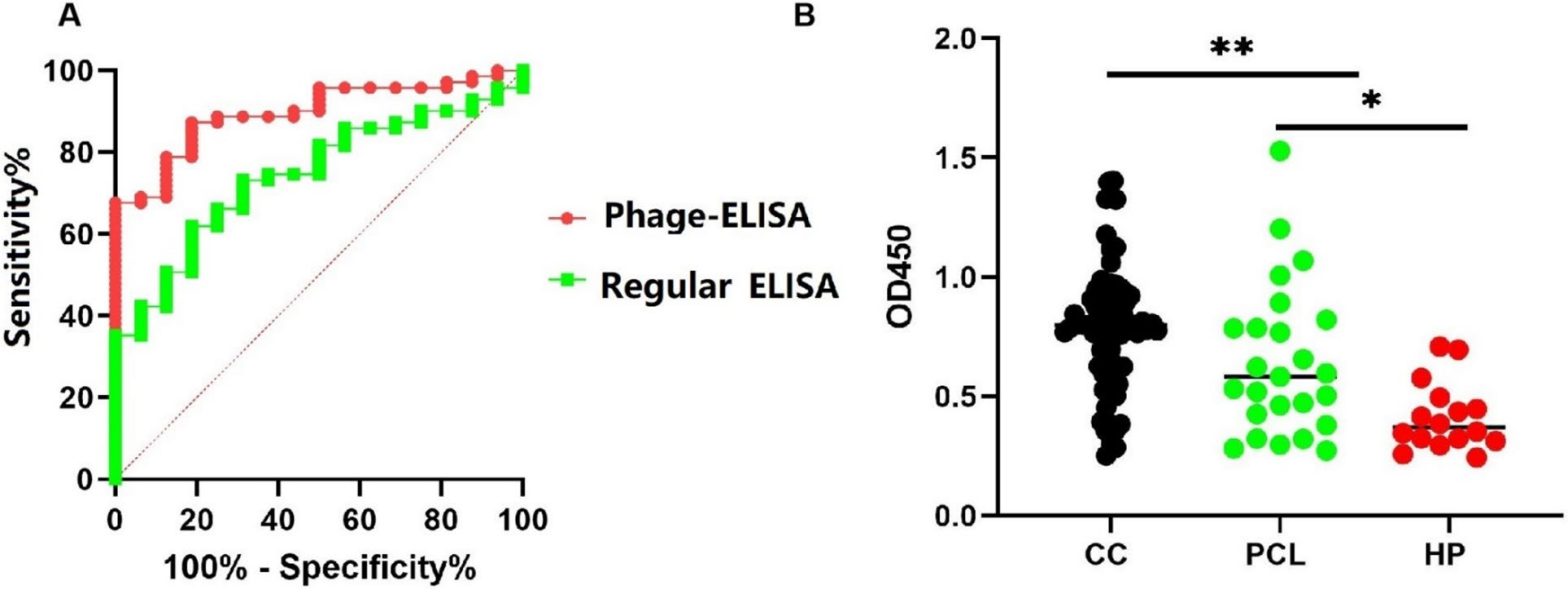
Detection of serum VEGF and sPD-L1 by phage sandwich ELISA method. Sixteen healthy human sera samples (HP), 25 precancerous cervical lesion samples (PCL), and 71 cervical cancer samples (CC) were studied in this article. **A** The area under the ROC curve (AUC) for serum VEGF and sPD-L1 was 0.896 when using the phage ELISA method in the cohort of 71 CC patients and 16 healthy controls. The area under the ROC curve (AUC) for serum VEGF and sPD-L1 was 0.826 when using regular sandwich ELISA (** *P* < *0.01*). **B** The OD450 in group of CC is significantly higher than the group of PCL and HP. (* *P* < 0.05, ** P < *0.01*)

In the sandwich phage ELISA assay, phage nanoparticles at a concentration of 50 μg/mL were employed as the coating agent. A mixture of anti-VEGF and anti-PD-L1 antibodies served as the detection antibodies. The phage nanofibers detected VEGF and sPD-L1 in serum samples with a positive rate of 78.9%. When anti-VEGF or anti-PD-L1 antibodies were used individually as detection antibodies, the positive rates for detecting VEGF alone and sPD-L1 alone in serum samples were 49.3% and 52.1%, respectively, with AUC values of 0.756 and 0.778 (*P* < *0.01*) (refer to Fig. S1 and Fig. S2). In comparison, using anti-VEGF or anti-PD-L1 antibodies alone as the coating agent resulted in only 42.3% and 49.3% of serum samples testing positive for each biomarker, respectively. This suggests that the fd phage nanofibers displaying peptides enhanced detection efficiency of biomarkers in cervical cancer patients. The AUC for serum VEGF and sPD-L1 was 0.896 when employing the phage ELISA method (Fig. 5), compared to 0.826 when using a combined measurement approach in the conventional sandwich ELISA method. This demonstrates the phage ELISA method’s strong diagnostic accuracy for cervical cancer biomarkers. Additionally, a significant difference was observed between the phage ELISA and conventional ELISA methods, with the phage ELISA method exhibiting higher sensitivity and specificity (*P* < *0.01*).

Overall, this study demonstrated that the phage ELISA method, employing dual-display fd nanofibers as a capture probe, represents a more sensitive and efficient approach for detecting VEGF and sPD-L1 in cervical cancer patients. ROC curve analysis further substantiated the diagnostic accuracy of this method.

## Discussions

Serum VEGF and sPD-L1 are recognized as predictive biomarkers for various types of cancer (Chivu-Economescu et al. 2023; Goncharuk et al. 2009; Jovanović et al. 2019; Mathur et al. 2005). In this study, serum VEGF and sPD-L1 levels were measured using conventional sandwich ELISA. Then, the cervical cancer group exhibited high concentrations of these markers, suggesting that serum VEGF and sPD-L1 may act as predictive biomarkers for cervical cancer. The combined measurement of these biomarkers holds potential applications in the diagnosis and management of cervical cancer (Arip et al. 2022; Liu et al. 2018; Yang et al. 2014). To enhance the sensitivity of diagnosing cervical cancer, the combined measurement of VEGF and sPD-L1 was employed. Our results suggest that the combination of serum VEGF and sPD-L1 can enhance the effectiveness of clinical diagnosis for cervical cancer, demonstrating an additive effect in diagnosing cervical cancer. Consequently, the combined measurement of VEGF and sPD-L1 could be a valuable tool for the early detection and diagnosis of cervical cancer.

*Fd phages*, specific to bacterial hosts, do not infect or harm mammalian cells and are remarkably stable under a range of environmental conditions, rendering them ideal for diagnostic applications (Wang et al. 2022). Furthermore, *fd phages* can be easily propagated and produced in large quantities, facilitating the development of cost-effective and scalable diagnostic assays (Daubie et al. 2022; González-Mora et al. 2020). Furthermore, *fd phage* can be engineered or selected to specifically target specific biomarkers (Parmley and Smith 1988). In previous research, two different peptides were displayed on the phage surface. This enabled the establishment of a phage ELISA method that replaces the need for combining the detection of two separate proteins (Wang et al. 2018). The study demonstrated the potential of genetically engineered *fd phage* nanofibers, with two different peptides displayed on their tip and side wall, for the diagnosis and prediction of cancer. In this study, *fd phage* nanofibers were engineered with peptides binding to VEGF and sPD-L1, allowing for the simultaneous detection of both proteins in a single assay. We displayed two functional peptides WT binding to PD-L1on one phage tip and peptide LC binding to VEGF along the sidewalls, and fd-LC-WT phage nanofibers demonstrated specific recognition of VEGF and PD-L1, respectively. This binding specificity of fd nanofibers thus enhances the accuracy and sensitivity of diagnostic assays. Consequently, the dual-display fd nanofibers can be utilized in diagnostic assays for accurate and sensitive detection of VEGF and sPD-L1 levels in patient samples.

To establish a reliable and efficient phage nanoparticle-based sandwich ELISA, various assay parameters were systematically optimized (Su et al. 2020). Concentrations of both capture and detection antibodies were systematically adjusted using a checkerboard titration method. Compared to conventional sandwich ELISA methods, the established phage-based sandwich ELISA proved to be more rapid and simpler, as phage nanofibers, being stable, can be engineered and mass-produced cost-effectively through bacteria infection in a short timeframe. Moreover, dual-display phage nanofibers, serving as multifunctional detection reagents, can be utilized to detect both single proteins and multiple proteins simultaneously, which can be utilized to detect individual cancer-related proteins, such as VEGF or sPD-L1, as demonstrated in previous study. When anti-VEGF antibodies were employed as detection antibodies, the phage ELISA method detects VEGF. Conversely, using a mixture of anti-VEGF and anti-PD-L1 antibodies as detection antibodies allowed for the simultaneous detection of both VEGF and sPD-L1 (Scheme 1).

In the analysis of serum samples from cancer patients, the phage ELISA method, employing dual-display phage nanofibers as a capture probe, demonstrated significantly higher detection sensitivity compared to conventional sandwich ELISA. Furthermore, the phage ELISA method is even more sensitive than the combined measurement approach of two conventional sandwich ELISAs. This suggests that the dual-displayed nanofibers have potential as diagnostic agents in cancer.

Therefore, the versatility of dual-display phage nanofibers enables the detection of individual proteins or the simultaneous detection of multiple proteins, rendering them a valuable tool for protein analysis and diagnosis across various fields, notably in cancer research. Additionally, these dual-display phage nanofibers have potential applications beyond cancer diagnosis, including predicting cancer progression and aiding in treatment strategies for cancer patients. The engineered phages have potential applications in cervical cancer immunotherapy, and they can function as targets for immune recognition, enabling the development of peptide-based vaccines that induce a specific anti-tumor immune response. Moreover, the phage display system allows for the screening of peptide libraries, facilitating the discovery of novel immunogenic peptides for therapeutic purposes. The phage displaying peptides could be employed in personalized cancer immunotherapies, where patient-specific peptides are selected based on unique tumor antigens. Overall, engineered phage play a crucial role in advancing cervical cancer immunotherapy.

## Supplementary Information

Below is the link to the electronic supplementary material.

Supplementary file1 (PDF 259 KB)

## Data Availability

Authors confirm that all relevant data are included in the article and/or its supplementary information files.
