# Supplementary material for: Advanced detection of cervical cancer biomarkers using engineered filamentous phage nanofibers
Source: Appl Microbiol Biotechnol. 2024 Feb 19;108:221. doi: 10.1007/s00253-024-13058-w (PMC10876719; doi:10.1007/s00253-024-13058-w)
Supplement: Supplementary file 1 [file 253_2024_13058_MOESM1_ESM.pdf]

Applied Microbiology and Biotechnology

Advanced Detection of Cervical Cancer Biomarkers Using Engineered Filamentous Phage Nanofibers

Xu Zhou[1],Yicun Wang2[†], Meijing Bao[1],Yuqing Chu[1], Ruixue Liu[1], Qi Chen[1], Yang Lin[1*†]

1 Department of Obstetrics and Gynecology, The Second Hospital of Jilin University, 1;

2 Jilin Provincial Key Laboratory on Molecular and Chemical Genetic, The Second Hospital of Jilin University, Changchun and 130041, China

* Correspondence:Yicun Wang, wangyicun@jlu.edu.cn

Lin Yang, linyang@jlu.edu.cn

Current address: 218 Ziqiang St, Changchun, JiLin, P.R. China,130041

† These authors contributed equally to this work.

Table S1. P/N value of Phage sandwich-ELISA checkerboard.

Capture antibody: fd-LC-WT	Detection antibody mix1	Mix 2	Mix 3	Mix 4	Mix 5	Mix 6	Mix 7	Mix 8	Mix 9	PBS
25ug/ml	2.87	2.71	2.82	2.86	2.81	2.96	2.75	2.48	2.29	0.92
50ug/ml	3.69	**3.91**	3.29	2.69	2.98	3.323	2.87	2.39	2.28	0.99
75ug/ml	3.05	3.34	3.66	3.05	3.28	3.29	2.81	2.75	2.43	0.99
100ug/ml	3.48	3.34	3.29	3.68	3.40	3.74	2.47	2.88	1.97	0.92

Table S2. The mixture of different dilutions of anti-VEGF and anti-PD-L1 antibodies

Dilution	anti-VEGF	1/2 500	1/ 5 000	1/ 10000
anti-PD-L1	1/2 500	Mix 1	Mix 2	Mix 3
	1/ 5 000	Mix 4	Mix 5	Mix 6
	1/ 10000	Mix 7	Mix 8	Mix 9

Table S3. Inter-batch reproducibility test of phage sandwich-ELISA.

Sample		OD450		cv%
P1	0.78	0.90	0.88	6.15
p2	0.67	0.79	0.73	7.11
p3	0.7	0.83	0.77	6.84
p4	1.17	1.48	1.27	9.92
p5	0.92	1.14	0.89	11.1
p6	0.74	0.88	0.81	7.38
N 1	0.36	0.44	0.43	9.01
N 2	0.28	0.3	0.33	10.04
N 3	0.40	0.54	0.47	11.58

*The repeatability of Phage ELISA kits was expressed as a coefficient of variation (CV) of results of sera tested 3 times in different days by the period of 1 month, by the same samples (serum from 6 cervical cancer patients and 3 healthy controls), in the same conditions, and by using the same equipment.

Table S4. Intra-batch reproducibility test of phage sandwich-ELISA.

Sample		**OD450**		**cv%**
P1	0.78	0.80	0.76	2.48
p2	0.67	0.73	0.74	4.41
p3	0.69	0.69	0.72	2.15
p4	1.17	0.98	0.99	8.36
p5	0.92	0.87	0.88	2.45
p6	0.74	0.69	0.75	3.56
N 1	0.36	0.34	0.36	3.56
N 2	0.28	0.3	0.29	3.07
N 3	0.40	0.37	0.39	4.95

* The repeatability of Phage ELISA kits was expressed as a coefficient of variation (CV) of results of sera tested 3 times in same day, by the same samples, in the same conditions, and by using the same equipment.

Figure S1. Detection of serum sPD-L1 by Phage sandwich-ELISA Method. 16 healthy human sera samples (HP), 25 precancerous cervical lesion samples (PCL）, and 71 cervical cancer samples (CC) were studied in this article. (A) The concentration of sPD-L1 in group of CC is significantly higher than the group of PCL and HP. (* *P < 0.05,* ** *P < 0.01*).; (B) The area under the ROC curve (AUC) for serum sPD-L1 was 0.778 when using the phage-ELISA method in the cohort of 71 CC patients and 16 healthy controls. (** *P < 0.01*).

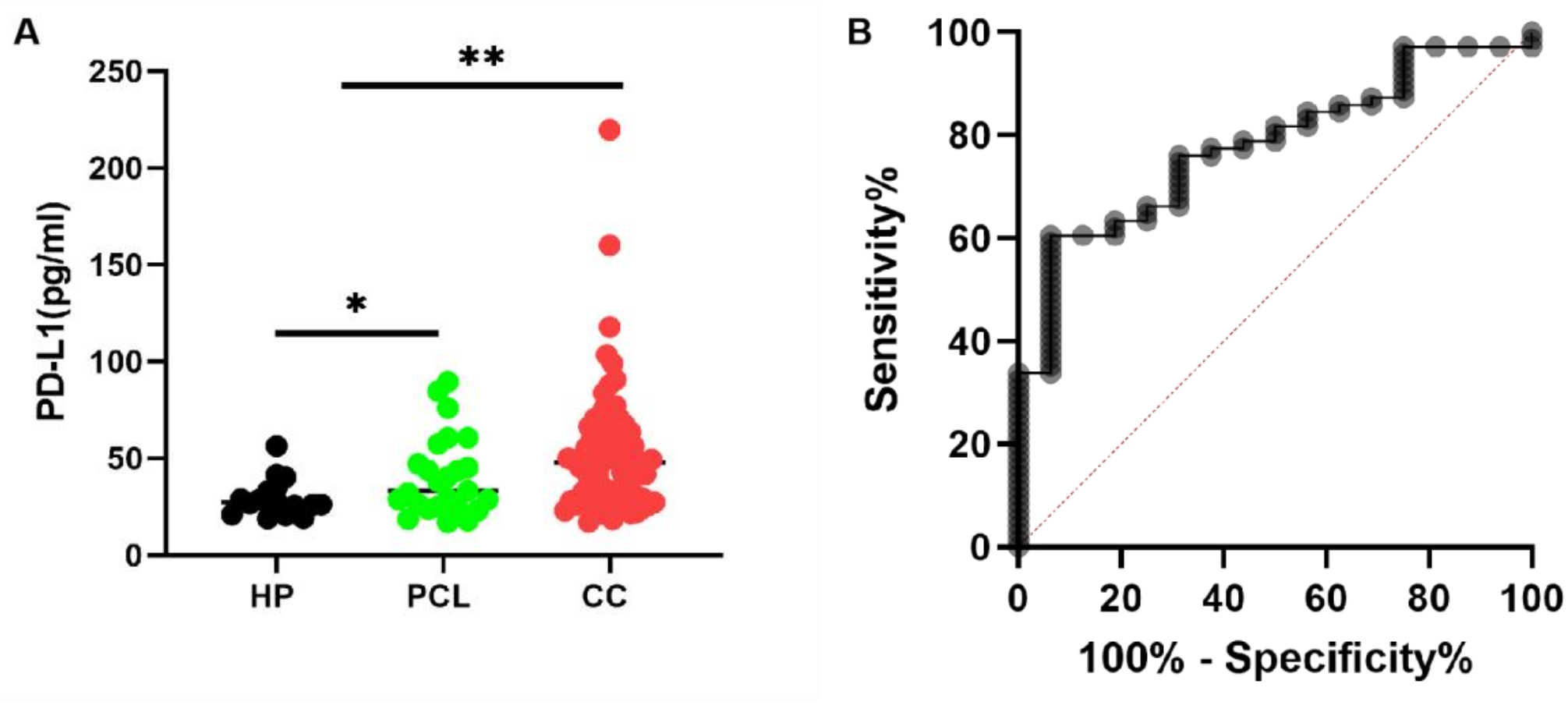

Figure S2. Detection of serum VEGF by Phage sandwich-ELISA Method. 16 healthy human sera samples (HP), 25 precancerous cervical lesion samples (PCL）, and 71 cervical cancer samples (CC) were studied in this article. (A) The concentration of VEGF in group of CC is significantly higher than the group of HP. (* *P < 0.05,* ** *P* < 0.01).; (B) The area under the ROC curve (AUC) for serum VEGF was 0.756 when using the phage-ELISA method in the cohort of 71 CC patients and 16 healthy controls. (** *P < 0.01*).

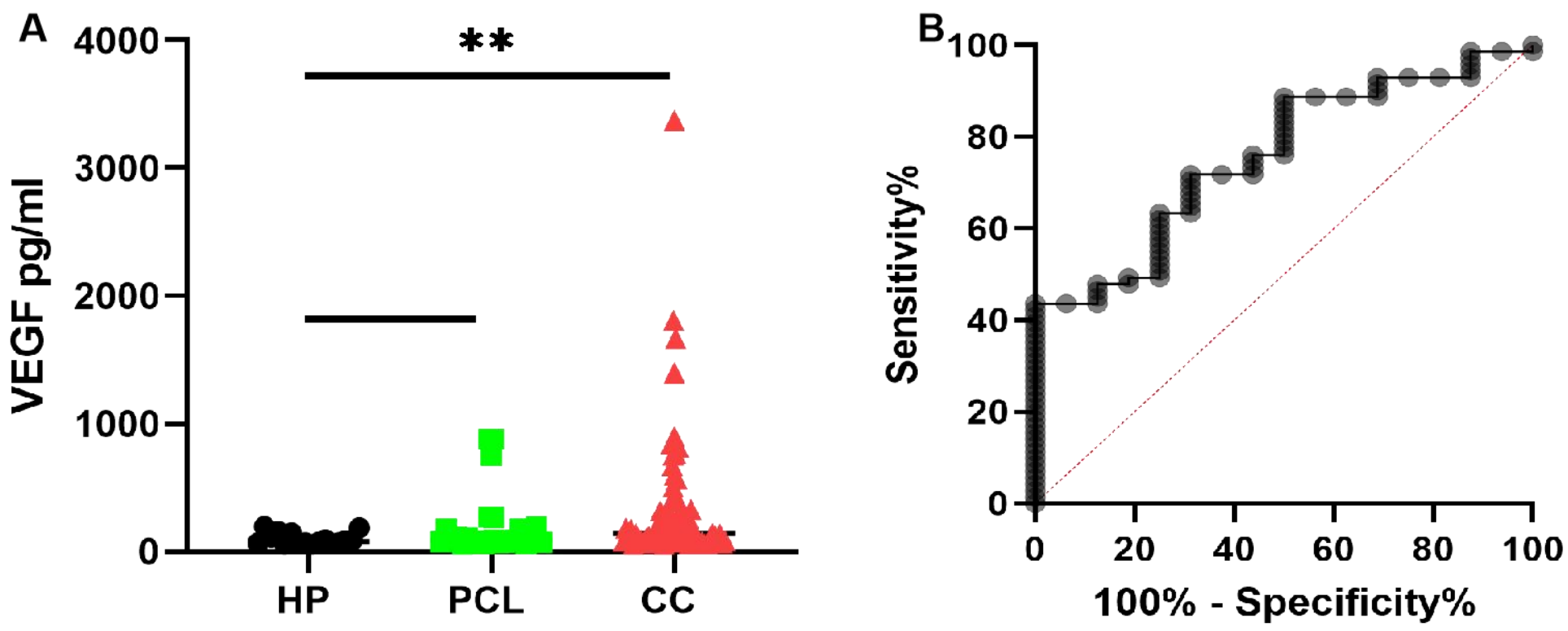

A
4000
3000
2000
1000
0
VEGF pg/ml
**
HP
PCL
CC
B
100
80
60
40
20
0
Sensitivity%
0
20
40
60
80
100
100% - Specificity%
